# Molecular Design of HER3-Targeting Affibody Molecules: Influence of Chelator and Presence of HEHEHE-Tag on Biodistribution of ^68^Ga-Labeled Tracers

**DOI:** 10.3390/ijms20051080

**Published:** 2019-03-02

**Authors:** Charles Dahlsson Leitao, Sara S. Rinne, Bogdan Mitran, Anzhelika Vorobyeva, Ken G. Andersson, Vladimir Tolmachev, Stefan Ståhl, John Löfblom, Anna Orlova

**Affiliations:** 1 Department of Protein Science, School of Engineering Sciences in Chemistry, Biotechnology and Health, KTH Royal Institute of Technology, 106 91 Stockholm, Sweden; chdl@kth.se (C.D.L.); ken2@kth.se (K.G.A.); ssta@kth.se (S.S.); lofblom@kth.se (J.L.); 2 Department of Medicinal Chemistry, Uppsala University, 751 23Uppsala, Sweden; sara.rinne@ilk.uu.se (S.S.R.); mitran.bogdan@ilk.uu.se (B.M.); 3 Department of Immunology, Genetics and Pathology, Uppsala University, 751 85Uppsala, Sweden; anzhelika.vorobyeva@igp.uu.se (A.V.); Vladimir.tolmachev@igp.uu.se (V.T.); 4 Science for Life Laboratory, Uppsala University, 751 23 Uppsala, Sweden

**Keywords:** HER3, affibody, NOTA, NODAGA, molecular imaging, gallium-68, PET

## Abstract

Affibody-based imaging of HER3 is a promising approach for patient stratification. We investigated the influence of a hydrophilic HEHEHE-tag ((HE)_3_-tag) and two different gallium-68/chelator-complexes on the biodistribution of Z_08698_ with the aim to improve the tracer for PET imaging. Affibody molecules (HE)_3_-Z_08698_-X and Z_08698_-X (X = NOTA, NODAGA) were produced and labeled with gallium-68. Binding specificity and cellular processing were studied in HER3-expressing human cancer cell lines BxPC-3 and DU145. Biodistribution was studied 3 h p.i. in Balb/c nu/nu mice bearing BxPC-3 xenografts. Mice were imaged 3 h p.i. using microPET/CT. Conjugates were stably labeled with gallium-68 and bound specifically to HER3 in vitro and in vivo. Association to cells was rapid but internalization was slow. Uptake in tissues, including tumors, was lower for (HE)_3_-Z_08698_-X than for non-tagged variants. The neutral [^68^Ga]Ga-NODAGA complex reduced the hepatic uptake of Z_08698_ compared to positively charged [^68^Ga]Ga-NOTA-conjugated variants. The influence of the chelator was more pronounced in variants without (HE)_3-_tag. In conclusion, hydrophilic (HE)_3_-tag and neutral charge of the [^68^Ga]Ga-NODAGA complex promoted blood clearance and lowered hepatic uptake of Z_08698_. [^68^Ga]Ga-(HE)_3_-Z_08698_-NODAGA was considered most promising, providing the lowest blood and hepatic uptake and the best imaging contrast among the tested variants.

## 1. Introduction

The human epidermal growth factor receptor type 3 (HER3) is overexpressed in over 40% of solid malignant tumors, among others in cervical and ovarian cancers, colorectal, gastric, breast, and prostate cancer [1,2]. In recent years, it has become evident that HER3 overexpression and oncogenic signaling is a major cause for therapy resistance and tumor progression [3,4]. HER3 is unique compared to the other members of the human epidermal growth factor receptor (HER) family in its non-functional tyrosine kinase domain [5]. As a result, heterodimerization with other members of the HER-family is required to initiate oncogenic downstream signaling [5]. HER3 is known to have a compensatory function in HER-signaling and the status of HER3 expression in cancer is often dynamic [6]. It is suggested that blocking of HER3 and its downstream signaling is needed to overcome therapy resistance [3,4]. Thus, HER3 has become an important target for cancer therapy. Several HER3-tageting therapeutic agents are currently being investigated in clinical trials, and more are in pre-clinical development stages [7]. However, it is necessary to be able to reliably identify patients with the corresponding molecular profile for HER3-targeted therapy to effectively treat patients and to avoid overtreatment.

Due to its non-invasive and repeatable nature, radionuclide molecular imaging is a promising alternative to biopsies for the detection of target expression in the body. Furthermore, radionuclide molecular imaging could not only be used for predicting response and to aid treatment planning, but would also allow to monitor the molecular development of the disease and assessment of treatment response. This is particularly beneficial in the case of HER3, because its expression can change during the course of treatment [6,8]

Comparably low levels of overexpression in tumors and natural expression of HER3 in healthy organs constitute specific challenges to HER3 imaging and require high affinity and specificity of the targeting molecules [9]. Monoclonal antibodies for PET imaging of HER3 expression have been investigated in preclinical and clinical settings [10,11]. A recent study using the zirconium-89 labeled monoclonal antibody lumretuzumab reported successful quantification of HER3-specific tracer uptake in cancer patients [10]. However, the authors indicated that enhanced permeability and retention effect (EPR) and high hepatic uptake due to endogenous expression and hepatobiliary excretion may limit the imaging contrast. In fact, none of the detected HER3 positive lesion could surpass the liver in tracer uptake [10]. In particular for targets with low levels of overexpression, the EPR effect and prolonged residence in blood, can limit the utility of antibodies for imaging [12]. This suggests that they may not be ideal candidates for imaging of HER3 expression.

We have previously proposed the use of affibody molecules for imaging of HER3 expression and demonstrated their ability to identify HER3 expression in tumors in pre-clinical models [13,14,15]. Affibody molecules are small (7–8 kDa) high-affinity scaffold proteins and promising imaging and therapeutic agents [16]. For example, HER2-status of breast cancer patients was accurately quantified by PET imaging using the gallium-68 labeled affibody molecule ABY-025 [17].

The results from previous studies on HER3-targeting affibody molecules performed by our and other groups are encouraging. The anti-HER3 affibody molecule Z_08698_ has been used successfully for imaging of HER3 expression in pre-clinical models with PET radionuclides gallium-68, fluorine-18 (via AlF chemistry), radiocobalt, and zirconium-89, as well as the SPECT radionuclide indium-111 [13,15,18,19,20]. HER3 expressing xenografts could be visualized as early as 1 h p.i. [18] and differentiation between high and low expressing xenografts was possible [15]. While the data are promising, the studies indicate that further optimization is required. Endogenous expression in healthy organs, first and foremost in liver, reduces the image contrast and would hence complicate image interpretation. It was hypothesized that hepatic uptake of affibody molecules is mediated by two separate mechanisms [20], where one is HER3-mediated and the other is unspecific and possibly influenced by hydrophilicity and local charge of the targeting molecule.

General approaches to reduce hepatic uptake include increasing the hydrophilicity via a hydrophilic chelator or linker, modifying the positioning and composition of potential purification tags or increasing (local) negative charge [21]. Studies with HER2-targeting affibody molecules demonstrated that the introduction of a hydrophilic and negatively charged HEHEHE-tag ((HE)_3_-tag) at the N-terminus together with a ^99m^Tc(CO)_3_ label reduced the hepatic uptake by up to 10-fold, thereby improving the imaging contrast tremendously [22,23].

Recent data on the influence of charge of the radiometal/chelator complex on the hepatic uptake of anti-HER3 affibody molecules further highlighted that increasing the negative charge on the C-terminus of the molecules improves the imaging contrast in the liver. For example, labeling of the anti-HER3 affibody molecule Z_08698_ with radiocobalt using NOTA as a chelator, resulted in a neutral cobalt-chelator complex, and appreciably lower hepatic uptake and a better tumor-to-liver ratio [20]. Furthermore, when the positively charged [^111^In]In-NOTA-complexwas exchanged with the negatively charged [^111^In]In-DOTAGA-complex, hepatic uptake was reduced 2-fold without influencing the tumor uptake [24]. The influence of different parameters affecting the hepatic uptake must therefore be carefully evaluated to optimize the imaging properties of anti-HER3 affibody molecules, particularly for increasing the imaging contrast in the liver. 

The rapid kinetics of affibody molecules allow for imaging at early timepoints making them compatible with shorter-lived radionuclides such as fluorine-18 (t_1/2_ = 110 min) or gallium-68 (t_1/2_ = 68 min). We have previously shown the possibility for PET-imaging of HER3 expression using a gallium-68 labeled affibody [15]. Generator-produced gallium-68 is available in clinics and we anticipate that by further improving the imaging properties of gallium-68 labeled anti-HER3 affibody molecules, the potential utility of this tracer for patient stratification could be enhanced. The triaza macrocyclic chelator NOTA was selected for this study, because it is considered to be the “golden standard” for labeling with ^68^Ga due to favorable labeling conditions and excellent in vivo stability [25]. NODAGA (a NOTA-derivative) offers a similar high-stability complex, but provides an extra negative charge of the complex due to presence of an additional carboxyl group. Using both NOTA and NODAGA, thus enables investigation of the influence of local charge on the labeled protein and its imaging properties.

The aims of this study were to investigate the influence of 1) hydrophilic and negatively charged histidine-glutamate-tag ((HE)_3_-tag) at the N-terminus, and 2) the charge of gallium-68 complex with different NOTA-derivatives at the C-terminus on the biodistribution of Z_08698_ and to apply this information for optimizing molecular design of affibody-based imaging probes.

## 2. Results

### 2.1. Tracer Production and Characterization

Four variants of the anti-HER3 affibody molecule Z_08698_ were produced: two (HE)_3_-conjugated variants ((HE)_3_-Z_08698_-X) and two non-tagged variants (Z_08698_-X), with X = NOTA, NODAGA. Production and characterization of (HE)_3_-Z_08698_-NOTA, Z_08698_-NOTA, and Z_08698_-NODAGA was previously described in [13,24] and data is presented in supplementary data for comparison along with the obtained results from production and characterization of (HE)_3_-Z_08698_-NODAGA. For (HE)_3_-Z_08698_-NODAGA, the (HE)_3_-Z_08698_ affibody was produced in *E. coli* and then site specifically conjugated to maleimide derivatives of NODAGA.

Purity was determined using Reverse-Phase High Perfromance Liquid Chromatography (RP-HPLC) and exceeded 95% for all conjugates (Figure S1, Table S1). Molecular masses (Table S1) were determined with electrospray ionization mass spectrometry (ESI-MS) (Figure S2) showing no discrepancy between experimental and theoretical values. Circular dichroism was used to measure thermal stability, refolding capacity, and associated melting temperatures (Figure S3 and Table S1). Kinetic data acquired from SPR analysis corresponded to K_D_ values in the low picomolar range (Table S1). The K_D_ value refers to the monovalent affinity for human HER3 according to a Langmuir 1:1 model. The representative sensorgrams with fitted curves are shown in Figure S4.

### 2.2. Radiolabeling

Radiolabeling was done in ascorbic acid buffer (1 M, pH 3.6). Conjugates were incubated for 15 min at 85 °C with 150–200 MBq gallium-68 eluate from the ^68^Ga/^68^Ge generator. Radiochemical yields were analyzed with instant thin layered chromatography (ITLC). Radiolabeling of Z_08698_-NODAGA and (HE)_3_-Z_08698_-NODAGA resulted in almost quantitative yields of 98 ± 1% and 97 ± 2% respectively (Table 1). Labeling of Z_08698_-NOTA resulted in 88 ± 11% radiochemical yield. Purity of all conjugates was above 98% after purification with NAP5 size exclusion columns. Release of the radiolabel was 1% or less for all conjugates when challenged in PBS or human serum for 1 h (Table 1). Radiolabeling of (HE)_3_-Z_08698_-NOTA for biodistribution yielded 87 ± 3% and a purity of 99.6 ± 0.5%. Stability of [^68^Ga]Ga-(HE)_3_-Z_08698_-NOTA was previously confirmed [15].

### 2.3. In Vitro Characterization

**Figure 1:**
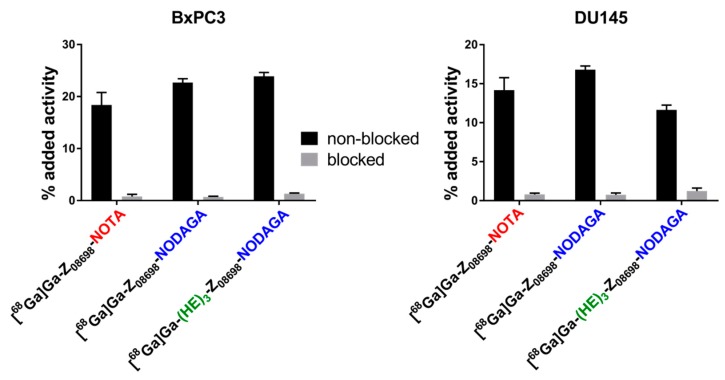
In vitro specificity test. HER3 receptors in the blocked group were pre-saturated by addition of 500-fold molar excess of non-labeled anti-HER3 affibody molecule.

In vitro experiments were performed using HER3-expressing cell lines BxPC-3 and DU145. In vitro characterization of [^68^Ga]Ga-(HE)_3_-Z_08698_-NOTA was previously described by our group [15]. To prove specific binding of the conjugates to HER3, HER3 receptors were pre-saturated with 500-fold molecular excess of a non-labeled anti-HER3 affibody molecule, before incubation with the radiolabeled conjugates. Pre-saturation resulted in significantly decreased uptake of all radiolabeled conjugates in both cell lines (*p* < 0.05), demonstrating HER3-mediated binding (Figure 1).

**Figure 2:**
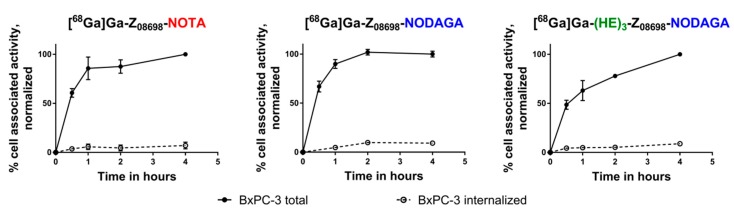
Cellular processing in BxPC-3 cells. Cells were continuously incubated with 0.1 nM of labeled construct at 37 °C. Error bars may not be visible because they are smaller than the curve symbols.

Cellular processing data from BxPC-3 cells are presented in Figure 2. Cells were continuously incubated with 0.1 nM of the radiolabeled conjugates. Membrane-bound activity was collected using a glycine buffer (pH 2) and remaining activity was considered internalized. Binding of the labeled constructs to the receptors was quick and internalization was slow. In BxPC-3 cells, 7–11% of maximum cell-associated radioactivity was internalized after 4 h continuous incubation. In DU145 cells (Figure S5), the internalized fractions were comparable to what was observed in BxPC-3 cells.

### 2.4. Biodistribution

For in vivo specificity test and biodistribution female Balb/c nu/nu mice bearing BxPC-3 xenografts were injected with 2 μg (700 kBq) [^68^Ga]Ga-(HE)_3_-Z_08698_-X or [^68^Ga]Ga-Z_08698_-X. Animals were sacrificed 3 h p.i., tumor and tissue samples were collected.

The in vivo specificity assay (Table 2) demonstrated that injection of excess amount non-labeled affibody molecule significantly decreased the tracer uptake in tumors and mErbB3 expressing organs (salivary glands, lungs, liver, stomach, and small intestine). Thus, binding of [^68^Ga]Ga-Z_08698_-NOTA, [^68^Ga]Ga-Z_08698_-NODAGA, and [^68^Ga]Ga-(HE)_3_-Z_08698_-NODAGA in vivo was HER3-specific.

Biodistribution pattern of all conjugates 3 h p.i. (Table 2) was characterized by low uptake in blood (between 0.1 and 0.3 %ID/g), elevated uptake in organs with natural expression of mErbB3 and high uptake in kidneys. The kidney uptake of [^68^Ga]Ga-(HE)_3_-Z_08698_-NODAGA was significantly higher compared to the other conjugates.

In contrast, more evident differences were observed between the variants without a (HE)_3_-tag. [^68^Ga]Ga-Z_08698_-NODAGA had significantly lower uptake in liver and spleen than [^68^Ga]Ga-Z_08698_-NOTA (Table 2). Activity concentration of [^68^Ga]Ga-Z_08698_-NOTA in blood was lower than for [^68^Ga]Ga-Z_08698_-NODAGA, however non-significant. In addition, tumor uptake of [^68^Ga]Ga-Z_08698_-NOTA was slightly higher than for [^68^Ga]Ga-Z_08698_-NODAGA, leading to a significantly higher tumor-to-blood ratio for [^68^Ga]Ga-Z_08698_-NOTA (Table 3).

Overall, [^68^Ga]Ga-(HE)_3_-Z_08698_-NODAGA presented the lowest liver uptake and the highest tumor-to-liver ratio together with high tumor-to-blood ratios.

### 2.5. Imaging

**Figure 3:**
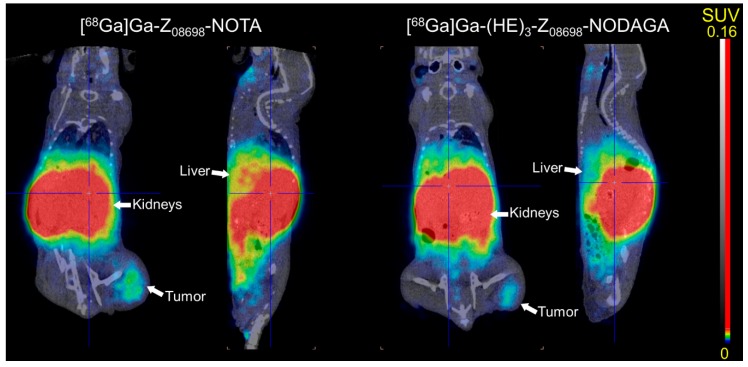
microPET-CT imaging of [^68^Ga]Ga-Z_08698_-NOTA (**left**) and [^68^Ga]Ga-(HE)_3_-Z_08698_-NODAGA (**right**) 3 h p.i. Mice bearing BxPC-3 xenografts were injected with 2 µg of the affibody molecules (4.8 MBq for [^68^Ga]Ga-Z_08698_-NOTA and 7.8 MBq for [^68^Ga]Ga-(HE)_3_-Z_08698_-NODAGA).

The results from the biodistribution were confirmed by micro PET/CT imaging (Figure 3). HER3 expressing xenografts could be clearly visualized. Furthermore, activity accumulation could be observed in mErbB3 expressing organs such as liver, in the GI tract and in the kidneys.

## 3. Discussion

It has previously been demonstrated that radiolabeled affibody molecules could be used to image HER3 expression in preclinical models [13,14,15,18], but further optimization of imaging contrast is still desirable. Many variables can influence the behavior of affibody-based imaging probes. Even small modifications in the molecular design, such as exchange of the radiometal, can have profound influence on its behavior [26]. There is thus an opportunity to further improve the biodistribution and imaging contrast of HER3-targeting affibody molecules by thoroughly investigating such variables. We hypothesized that increased hydrophilicity and exclusion of the positive charge of the gallium-68/chelator complex may improve the biodistribution and imaging contrast. For this reason, we designed and compared a total of four variants of the anti-HER3 affibody molecule Z_08698_, either with or without (HE)_3_-tag at the N-terminus and conjugated to NOTA or NODAGA chelator at the C-terminus.

Neither the (HE)_3_-tag nor the chelator had any influence on the stability of the label, binding specificity in vitro and in vivo or cellular processing in vitro. The pattern of internalization was typical for affibody molecules and characterized by rapid association but slow internalization rate. Even though the general pattern was comparable with earlier studies, the internalized fraction was lower than earlier reported for [^68^Ga]Ga-(HE)_3_-Z_08698_-NOTA [15]. It could be speculated that the excretion of catabolites is affected by the choice of the molecular design and radiometal-complex. However, internalization patterns for [^68^Ga]Ga-Z_08698_-NOTA and [^68^Ga]Ga-Z_08698_-NODAGA were in good agreement with published data for indium-111 labeled counterparts [24].

The biodistribution pattern of the tested molecules (Table 2) was characteristic for affibody molecules in general [13,15] and correlated with earlier published data for ^68^Ga-(HE)_3_-Z_08698_-NOTA [15]. This includes fast renal clearance from blood, a high level of renal reabsorption and elevated uptake in organs with endogenous expression of mErbB3, such as liver and GI-tract. Nevertheless, the results of this study demonstrated that the choice of chelator and presence or absence of the (HE)_3_-tag influenced the biodistribution of gallium-68 labeled anti-HER3 affibody molecules in vivo.

Presence of the (HE)_3_-tag at the N-terminus reduced the overall tissue uptake, including the tumors, compared to the variants without tag. The uptake of (HE)_3_-containing variants was approximately 1.5-fold lower in tumors because of more rapid clearance. Activity uptake in liver was close to 3-fold lower. Consequently, [^68^Ga]Ga-(HE)_3_-Z_08698_-NOTA and [^68^Ga]Ga-(HE)_3_-Z_08698_-NODAGA demonstrated superior tumor-to-liver ratios compared with [^68^Ga]Ga-Z_08698_-NOTA and [^68^Ga]Ga-Z_08698_-NODAGA despite lower tumor uptake. In the other mErbB3 expressing organs, the shift in uptake between tagged and non-tagged variants was nearly proportional to the shift in tumor and consequently no major differences in tumor-to-organ ratios were observed (Table 3).

These results were in agreement with previous studies, suggesting that increased hydrophilicity and presence of negatively charged amino acids reduces the hepatobiliary excretion or hepatic uptake and facilitates clearance of anti-HER2 affibody molecules [21,22,23]. In these previous studies on HER2-targeting affibody molecules, introduction of the (HE)_3_-tag caused a 5–10-fold reduction in hepatic uptake for indium-111 and technetium-99m labeled tracers, but had no effect on the tumor uptake [22,23]. This is in contrast to the results from this study, where presence of the (HE)_3_-tag showed an overall decreased uptake, including the tumors. It has to be noted that although the scaffold of the different affibody molecules is the same, the composition of the binding sites is different. The amino acids in the binding site constitute a substantial part of the surface, i.e., 13 out of 58 amino acid residues. Therefore, factors strongly affecting the biodistribution of one affibody molecule might be masked in another. Thus, the experience with anti-HER2 affibody molecules should be cautiously translated to anti-HER3 affibody molecules. Nevertheless, we could conclude that (HE)_3_-tagged variants are favorable to non-tagged variants for in vivo imaging. In fact, the tumor-to-liver ratio of [^68^Ga]Ga-(HE)_3_-Z_08698_-NODAGA was also superior to the reported ratios for Z_08698_ labeled with PET-nuclides zirconium-89 and radiocobalt 3 h p.i. [15,19].

The gallium-68/chelator complex influenced the uptake in normal organs but had no effect on tumor uptake. When comparing the non-tagged variants, [^68^Ga]Ga-Z_08698_-NODAGA had significantly lower hepatic uptake and a higher tumor-to-liver ratio than its NOTA-conjugated counterpart. Moreover, a similar trend was observed in other mErbB3 expressing organs, such as salivary glands, stomach, and intestines.

The major difference between the [^68^Ga]Ga-NOTA and [^68^Ga]Ga-NODAGA complexes is the overall charge, which is +1 and 0, respectively. The difference in structure is minor as both are triaza-chelators, and NODAGA is a derivative of NOTA with an additional carboxylic arm available for chelation [25,27]. Eliminating the positive charge of the [^68^Ga]Ga-NOTA complex in favor of the neutral charge of the [^68^Ga]Ga-NODAGA complex reduced the accumulation Z_08698_ in the liver. This is in agreement with previous studies on HER2 affibody molecules [23,28] and our recent experience with indium-111 labeled Z_08698_ [24]. Difference in complex charge for [^68^Ga]Ga-Z_08698_-NOTA and [^68^Ga]Ga-Z_08698_-NODAGA did not influence kidney activity uptake for the tested conjugates that had the same patter as for [^111^In]In-Z_08698_-NOTA and [^111^In]In-Z_08698_-NODAGA [24]. Addition of the (HE)_3_-tag to the conjugates in this study directed renal activity uptake oppositely: while renal uptake for NOTA-containing conjugate decreased about 2-fold, renal uptake for NODAGA-containing conjugate increased 1.5-fold. Because we still do not know the exact mechanism of renal uptake of affibody molecule (previously we were able to exclude cubulin/megalin pathway [29]) we can only speculate on the nature of this phenomenon. Local charge and lipophilicity patches may influence renal absorption and retention of radio catabolites in the kidneys.

Interestingly, presence of (HE)_3_-tag appeared to reduce the influence of the chelator on the biodistribution of Z_08698_. The only difference between biodistribution of [^68^Ga]Ga-(HE)_3_-Z_08698_-NODAGA and [^68^Ga]Ga-(HE)_3_-Z_08698_-NOTA was the faster blood clearance of [^68^Ga]Ga-(HE)_3_-Z_08698_-NODAGA, which resulted in almost 2-fold higher tumor-to-blood ratio. Hepatic uptake of [^68^Ga]Ga-(HE)_3_-Z_08698_-NODAGA was slightly but non-significantly lower than for [^68^Ga]Ga-(HE)_3_-Z_08698_-NOTA. Thus, the impact of the radiometal-chelator complex might be overshadowed by the presence of the hydrophilic histidine-glutamate-tag. It might be that the position of the modification (N- versus C-terminus) influences its effect. It has previously been speculated that N-terminal modifications have greater influence on the properties of HER2-targeting affibody than C-terminal modification due to the proximity to the binding site [23,30].

Both absolute tumor uptake and target-to-non-target ratios are important for the selection of the best imaging agent. In the present study, we have found that despite lower tumor uptake conjugate containing hydrophilic N-termini and neutrally charged C-termini preserved high tumor-to-blood ratio and had the best tumor-to-liver ratios.

In summary, the comparisons showed that the biodistribution of (HE)_3_-containing variants were more favorable than the biodistribution of non-(HE)_3_-containing variants, and that the use of a NODAGA chelator provided better biodistribution compared to the use of a NOTA chelator. However, the additional effect of simultaneous use of both modifications was minor. Still, [^68^Ga]Ga-(HE)_3_-Z_08698_-NODAGA was considered the most promising among the tested conjugates. The increase in hydrophilicity by introducing a (HE)_3_-tag at the N-terminus and eliminating the positive charge at the C-terminus by using a NODAGA chelator lead to the fastest clearance from blood and best tumor-to-liver ratio, resulting in improved contrast for imaging of HER3-expression using gallium-labeled anti-HER3 affibody molecules. The results of this study should be taken into account in the molecular design of affibody-based imaging probes.

## 4. Materials and Methods

HER3 expressing human cancer cell lines BxPC3 (pancreatic cancer) and DU145 (prostate cancer) from ATCC (American Type Culture Collection) were cultured in RPMI-1640 media (Biochrom, Berlin, Germany) supplemented with 10% Fetal Bovine Serum (Merck, Darmstadt, Germany), 1% penicillin-streptomycin, and 1% L-glutamine (both Biochrom, Berlin, Germany). A 3-inch NaI(Tl) detector (1480 Wizard; Wallac Oy, Turku, Finland) was used to measure the radioactivity content in samples. Measured activity was corrected for decay and data are displayed as average with standard deviation. Unpaired, two-tailed *t*-test was used to determine statistical significance (*p* < 0.05) for all in vitro and in vivo specificity experiments. To test for statistically significant differences between the uptake of the conjugates in the biodistribution study one-way ANOVA with multiple comparisons with Bonferroni correction was used in GraphPad Prism (GraphPad Software, San Diego, CA, USA).

All animal studies were approved by the Ethics Committee for Animal Research in Uppsala, Sweden (ethical permission C5/16 from 26-02-2016) and followed the national legislation on protection of laboratory animals.

### 4.1. Tracer Production and Characterization

Production and characterization of (HE)_3_-Z_08698_-NOTA, Z_08698_-NOTA, and Z_08698_-NODAGA has been previously described in [13,24]. Production, conjugation, purification, and characterization of (HE)_3_-Z_08698_-NODAGA was performed as follows.

The (HE)_3_-tagged HER3-binding affibody (HE)_3_-Z_08698_ was produced in BL21*(DE3) *E. coli* (Thermo Fisher Scientific, Watham, MA, USA) in an overnight culture at 25 °C after induced expression with 100 μM IPTG at an OD_600nm_ of 0.8. Following cell lysis with French press, the supernatant was heated to 90 °C for 10 min with subsequent incubation on ice for 20 min whereupon the aggregates were spun down as a first-step removal of unwanted proteins. The affibody molecule was recovered on an ÄKTAexplorer (GE Healthcare, Uppsala, Sweden) using a 3 mL Ni Sepharose 6 Fast Flow column (GE Healthcare). The buffer of the eluate was changed to 20 mM NH_4_Ac (pH 5.5) and the proteins were freeze-dried.

The protein was dissolved in 20 mM NH_4_Ac (pH 5.5) and reduced with a molar concentration of tris(2-carboxyethyl)phosphine (TCEP) equal to the protein concentration for 30 min at 37 °C. The (HE)_3_-Z_08698_ affibody was incubated at 37 °C for 90 min with 10-fold molar excess of maleimide derivative NODAGA (CheMatech) for site-specific conjugation to the C-terminal cysteine on the affibody. Metal ion contaminations were removed from all buffers used with Chelex 100 resin (Bio-Rad Laboratories, Hercules, CA, USA).

After the site-specific conjugation, reverse-phase high performance liquid chromatography (RP-HPLC) on a 1200 series HPLC system using a Zorbax 300SB-C18 semi-preparative column (Agilent Technologies, Santa Clara, CA, USA) was used for purification. Water with 0.1% trifluoroacetic acid was used as running buffer and an acetonitrile gradient was used for elution.

### 4.2. Characterization

The purity was determined using RP-HPLC and an analytical Zorbax 300SB-C18 column (Agilent Technologies). Circular dichroism spectroscopy was performed using a Chirascan spectropolarimeter (Applied Photophysics, United Kingdom) with an optical path length of 1 mm, to analyze the alpha-helical content, thermal stability and refolding capacity of (HE)_3_-Z_08698_-NODAGA at a concentration of 0.25 mg/mL. The thermal stability was evaluated by measuring the change in ellipticity at 221 nm during heating (5 °C/min) from 20 to 90 °C. The melting temperature (T_m_) was approximated from the data acquired from the variable temperature measurements (VTM) by curve fitting using a Boltzmann Sigmoidal model (GraphPad Prism, version 7, La Jolla, CA, USA). The refolding capacity was assessed by comparing spectra obtained from measurements at wavelengths in the range 195–260 nm at 20 °C, before and after thermal denaturation.

Electrospray ionization mass spectrometry (ESI-MS) with a 6520 Accurate-Mass Q-TOF LC/MS (Agilent Technologies) was used to confirm molecular mass of the purified conjugate.

The affinity to human HER3 was investigated using surface plasmon resonance (SPR). Single-cycle kinetics on a Biacore T200 system (GE Healthcare, Chicago, IL, United States) using a CM5 sensor chip with immobilized human HER3-Fc (Sino Biological) was used for the analysis. Five concentrations (3.125, 6.25, 12.5, 25, and 50 nM) of (HE)_3_-_08698_-NODAGA were sequentially injected in a single cycle with a contact time of 150 s for each concentration. The acquired sensorgram was analyzed using a Langmuir 1:1 kinetic model.

### 4.3. Labeling and In Vitro Stability

Gallium-68 was obtained by elution of a ^68^Ga/^68^Ge-generator (Cyclotron Co. Obninsk, Russia) with 0.1 M HCl. Eluate was collected in fractions of 400 µl at a speed of 800 µl/min. The third fraction containing in average 452 ± 147 MBq was used for radiolabeling.

All conjugates were radiolabeled according to the following protocol. Solution of 25 µg of affibody molecule in 300 µL ascorbic acid buffer (1 M, pH 3.6) was incubated with 200 µl of gallium-68 eluate (150-200 MBq) for 15 min at 85 °C. Labeling yields were determined with ITLC (Instant Thin Layered Chromatography). Samples were applied to silica gel-impregnated glass microfiber chromatography paper (Agilent Technologies, Santa Clara, CA, USA) and eluted with 0.2 M citric acid. If necessary, the radiolabeled conjugates were purified with NAP-5 size-exclusion columns (GE Healthcare), pre-washed with 1% BSA/PBS.

To test the stability, 2 µg of labeled conjugates were incubated for 1 h in PBS at room temperature or in human serum at 37 °C. The samples were analyzed with ITLC.

### 4.4. In Vitro Characterization

In vitro characterization of [^68^Ga]Ga-(HE)_3_-Z_08698_-NODAGA [^68^Ga]Ga-Z_08698_-NOTA and [^68^Ga]Ga-Z_08698_-NODAGA was performed in BxPC-3 (pancreatic cancer, 12 × 10^3^ receptors/cell) and DU145 (prostate cancer, 6 × 10^3^ receptors/cell) as previously described [15]. [^68^Ga]Ga-(HE)_3_-Z_08698_-NOTA has been previously characterized [15] and was therefore not included. Cells were seeded one day prior to the experiments in 3 cm petri dishes with a density of 10^6^ cells/dish. Triplicates were used for each data point.

For testing of binding specificity, two sets of dishes were used. In one set, HER3 receptors were pre-saturated with 50 nM of non-radiolabeled anti-HER3 affibody molecules. Cells were incubated with 0.1 nM of the labeled conjugates for 1 h at 37 °C. Afterwards, incubation media and cells were collected and samples were measured in the automated gamma counter.

To study cellular processing of the conjugates, BxPC-3 cells were continuously incubated with 0.1 nM of labeled conjugate for up to 4 h. DU145 cells were incubated up to 2 h. At selected timepoints, the incubation media, the membrane bound and internalized fraction of the conjugates were collected. Membrane-bound activity was collected after incubation with 0.2 M glycine buffer containing 0.15 M NaCl, 4 M Urea, pH 2, for 5 min on ice. Thereafter, cells were incubated with 1 M NaOH for 30 min at 37 °C to collect the internalized activity. Samples were measured in the automated gamma counter.

### 4.5. In Vivo Biodistribution

Biodistribution of [^68^Ga]Ga-Z_08698_-NOTA, [^68^Ga]Ga-(HE)_3_-Z_08698_-NODAGA and [^68^Ga]Ga-Z_08698_-NODAGA was studied at 3 h p.i. in female Balb/c nu/nu mice carrying BxPC-3 xenografts. The previously studied [^68^Ga]Ga-(HE)_3_-Z_08698_-NOTA [15] was included as a reference.

Mice were implanted with BxPC-3 (5 × 10^6^/mouse) 20 days prior to the experiment. At time of the experiment, tumors weighed 0.06 ± 0.02 g and average mouse weight was 18.3 ± 0.6 g.

Per compound, a group of four mice was injected intravenously with 2 µg labeled affibody molecule (700 kBq, in 100 µL 1% BSA/PBS). At 3 h p.i., animals were sacrificed after pre-injection of Ketalar-Rompun anesthetic solution (10 mg/mL Ketalar and 1 mg/mL Rompun; 20 μL solution/gram of body weight). Samples of blood, salivary glands, lungs, liver, stomach, small intestines, spleen, kidneys, tumor, muscle, and bone were collected, weighed, and measured in the automated gamma counter. Content of radioactivity was calculated as percent injected activity per gram (%ID/g). Carcass and GI tract were collected and uptake was calculated as %ID.

To demonstrate specific uptake in vivo, the protein dose was adjusted to 70 µg/mouse (700 kBq) using non-labeled affibody molecule. A biodistribution was performed 3 h p.i. according to the protocol described above. Receptor mediated binding of [^68^Ga]Ga-(HE)_3_-Z_08698_-NOTA was previously demonstrated in vivo [15].

### 4.6. Imaging

Whole body positron emission tomography (PET)/computed tomography (CT) scans of the BxPC-3 xenografted mice injected with [^68^Ga]Ga-Z_08698_-NOTA (2 µg, 4.8 MBq) and [^68^Ga]Ga-(HE)_3_-Z_08698_-NODAGA (2 µg, 7.8 MBq) were performed in a Triumph Trimodality PET/SPECT/CT System (TriFoil Imaging, Inc., Northridge, CA, USA) at 3 h p.i. The animals were euthanized by CO2 asphyxiation immediately before the scans. PET acquisitions were performed for 60 min (8 cm field of view (FOV)). CT acquisitions were performed at the following parameters: 8 cm FOV; 1.48 magnification; one projection; 512 frames for 2.13 min. PET data were reconstructed into a static image using an ordered subsets expectation maximization 3D algorithm (20 iterations). CT raw data were reconstructed using filtered back projection. PET and CT data were fused and analyzed using PMOD v3.510 (PMOD Technologies Ltd., Zurich, Switzerland). Coronal, axial, and sagittal PET-CT images were presented on RGB color scale.

## Figures and Tables

**Table 1:** Labeling and in vitro stability. Average radiochemical yield (*n* = 4–7) and purity of conjugates after NAP5 size-exclusion purification. To test stability, 2 µg of the purified conjugates was incubated for 1 h in PBS orhuman serum. Stability is presented as % release.

Type of analysis	[^68^Ga]Ga-Z_08698_-NOTA (*n* = 4)	[^68^Ga]Ga-Z_08698_-NODAGA (*n* = 7)	[^68^Ga]Ga-(HE)_3_-Z_08698_-NODAGA (*n* = 4)
Labeling Yield, %	88 ± 11 ^a,b^	98 ± 1 ^a^	97 ± 2 ^b^
Purity after purification, %	98.9 ± 0.6 ^b^	99.6 ± 0.1	99.8 ± 0.06 ^b^
Release in PBS (1 h, RT), %	0.9 ± 0.2 ^b^	0.7 ± 0.2 ^c^	0.03 ± 0.05 ^b,c^
Release in human serum (1 h, 37 °C), %	1.2 ± 0.5 ^b^	1.1 ± 0.1 ^c^	0.23 ± 0.05 ^b,c^

Statistical analysis was performed using one-way ANOVA with multiple comparisons using Bonferroni correction. Differences were significant (*p* < 0.05) between ^a^ [^68^Ga]Ga-(HE)_3_-Z_08698_-NOTA and [^68^Ga]Ga-Z_08698_-NODAGA, ^b^ [^68^Ga]Ga-Z_08698_-NOTA and [^68^Ga]Ga-(HE)_3_-Z_08698_-NODAGA, ^c^ [^68^Ga]Ga-Z_08698_-NODAGA and [^68^Ga]Ga-(HE)_3_-Z_08698_-NODAGA.

**Table 2:** In vivo biodistribution and specificity 3 h p.i. as %ID/g. Female Balb/c nu/nu mice with BxPC-3 xenografts were injected with 2 µg of labeled affibody conjugate. For specificity test, protein dose was adjusted to 70 µg using non-labeled HER3-targeting affibody molecule. In vivo specificity of [^68^Ga]Ga-(HE)_3_-Z_08698_-NOTA was previously demonstrated [15].

Organ	[^68^Ga]Ga-(HE)_3_-Z_08698_-NOTA	[^68^Ga]Ga-Z_08698_-NOTA		[^68^Ga]Ga-(HE)_3_-Z_08698_-NODAGA		[^68^Ga]Ga-Z_08698_-NODAGA	
	2 µg	2 µg	70 µg	2 µg	70 µg	2 µg	70 µg
Blood	0.22 ± 0.08	0.28 ± 0.04 ^e^	0.27 ± 0.02	0.126 ± 0.004 ^e,g^	0.11 ± 0.02	0.31 ± 0.06^g^	0.24 ± 0.03
Sal. Glands	0.9 ± 0.3 ^b,d^	2.2 ± 0.3 ^b,e^	0.5 ± 0.1 ^a^	1.0 ± 0.1 ^e,g^	0.35 ± 0.09 ^a^	1.8 ± 0.3 ^d,g^	0.40 ± 0.04 ^a^
Lungs	0.9 ± 0.3 ^b,d^	1.7 ± 0.1 ^b,e^	0.6 ± 0.1 ^a^	0.94 ± 0.06 ^e,g^	0.34 ± 0.06 ^a^	1.6 ± 0.2 ^d,g^	0.55 ± 0.09 ^a^
Liver	2.2 ± 0.7 ^b,d^	6.0 ± 0.6 ^b,e,f^	3.0 ± 0.6 ^a^	2.0 ± 0.2 ^e,g^	0.51 ± 0.04 ^a^	4.5 ± 0.6 ^d,f,g^	1.5 ± 0.2 ^a^
Spleen	0.3 ± 0.1	0.67 ± 0.06 ^e,f^	0.72 ± 0.09	0.3 ± 0.01 ^e^	0.21 ± 0.04	0.45 ± 0.6 ^f^	0.39 ± 0.06
Stomach	0.9 ± 0.4 ^b,d^	2.2 ± 0.3 ^b,e^	0.5 ± 0.2 ^a^	1.2 ± 0.2 ^e,g^	0.32 ± 0.05 ^a^	1.8 ± 0.4 ^d,g^	0.48 ± 0.07 ^a^
Small Intestine	2.0 ± 0.1 ^b,d^	6 ± 2 ^b,e^	0.8 ± 0.1 ^a^	2.7 ± 0.4 ^e,g^	0.4 ± 0.1 ^a^	5.1 ± 0.8 ^d,g^	0.7 ± 0.2 ^a^
Kidney	138 ± 28 ^b,c,d^	214 ± 22 ^b,e^	278 ± 44 ^a^	324 ± 11 ^c,e,g^	275 ± 48 ^a^	237 ± 24 ^d,g^	299 ± 43 ^a^
Tumor	1.9 ± 0.7 ^b,d^	3.9 ± 0.6 ^b,e^	1.1 ± 0.2 ^a^	1.9 ± 0.2 ^e,g^	0.7 ± 0.1 ^a^	3.3 ± 0.5 ^d,g^	0.9 ± 0.2 ^a^
Muscle	0.12 ± 0.08	0.19 ± 0.04	0.23 ± 0.10	0.11 ± 0.04	0.13 ± 0.05	0.18 ± 0.04	0.16 ± 0.05
Bone	0.32 ± 0.09	0.42 ± 0.08	0.4 ± 0.3	0.4 ± 0.2	0.19 ± 0.04	0.42 ± 0.1	0.4 ± 0.2
GI (% ID)	4 ± 1 ^b,d^	9.3 ± 0.4 ^b,e,f^	1.7 ± 0.8 ^a^	4 ± 1 ^e,g^	0.9 ± 0.4 ^a^	7 ± 1 ^d,f,g^	1.3 ± 0.6 ^a^
Body (% ID)	6 ± 2 ^b,d^	13 ± 3 ^b,e,f^	5.4 ± 0.7 ^a^	7 ± 1 ^e^	4.1 ± 1.0 ^a^	9.2 ± 0.8 ^d,f^	5.0 ± 0.8 ^a^

Statistical analysis was performed using one-way ANOVA with multiple comparisons using Bonferroni correction. Differences were significant (*p* < 0.05) between ^a^ 2 µg and 70 µg, ^b^ [^68^Ga]Ga-(HE)_3_-Z_08698_-NOTA and [^68^Ga]Ga*-Z_08698_-NOTA*, ^c^ [^68^Ga]Ga-(HE)_3_-Z_08698_-NOTA and [^68^Ga]Ga-(HE)_3_-Z_08698_-NODAGA, ^d^ [^68^Ga]Ga-(HE)_3_-Z_08698_-NOTA and [^68^Ga]Ga-Z_08698_-NODAGA, ^e^ [^68^Ga]Ga-Z_08698_-NOTA and [^68^Ga]Ga-(HE)_3_-Z_08698_-NODAGA, ^f^ [^68^Ga]Ga-Z_08698_-NOTA and [^68^Ga]Ga-Z_08698_-NODAGA, ^g^ [^68^Ga]Ga-(HE)_3_-Z_08698_-NODAGA and [^68^Ga]Ga-Z_08698_-NODAGA.

**Table 3:** Tumor-to-organ ratios 3 h p.i. Female balb/c nu/nu mice with BxPC-3 xenografts were injected with 2 µg of radiolabeled conjugate.

Organ	[^68^Ga]Ga-(HE)_3_-Z_08698_-NOTA	[^68^Ga]Ga-Z_08698_-NOTA	[^68^Ga]Ga-(HE)_3_-Z_08698_-NODAGA	[^68^Ga]Ga--Z_08698_-NODAGA
Blood	8.5 ± 0.6 ^a,b,c^	14 ± 2 ^a,e^	15 ± 2 ^b,f^	11 ± 1 ^e,f,c^
Salivary Glands	2.0 ± 0.1	1.8 ± 0.2	1.8 ± 0.4	1.9 ± 0.2
Lung	2.0 ± 0.2	2.3 ± 0.4	2.0 ± 0.1	2.0 ± 0.2
Liver	0.83 ± 0.10	0.64 ± 0.07 ^d^	1.0 ± 0.2 ^d^	0.8 ± 0.2
Spleen	5.5 ± 0.8	5.8 ± 0.6	8 ± 1	7.5 ± 0.9
Stomach	2.2 ± 0.2	1.8 ± 0.5	1.7 ± 0.3	1.8 ± 0.5
Small intestine	0.75 ± 0.08	0.8 ±0.4	0.7 ± 0.1	0.7 ± 0.1
Kidney	0.014 ± 0.002 ^b^	0.018 ± 0.002 ^d^	0.006 ± 0.001 ^b,d,f^	0.014 ± 0.003 ^f^
Muscle	16 ± 3	20 ± 2	14 ± 1	20 ± 4
Bone	6.0 ± 0.7	9 ± 1	7 ± 5	9 ± 3

Statistical analysis was performed using one-way ANOVA with multiple comparisons using Bonferroni correction. Differences were significant (*p* < 0.05) between ^a^ [^68^Ga]Ga-(HE)_3_-Z_08698_-NOTA and [^68^Ga]Ga-Z_08698_-NOTA, ^b^ [^68^Ga]Ga-(HE)_3_-Z_08698_-NOTA and [^68^Ga]Ga-(HE)_3_-Z_08698_-NODAGA, ^c^ [^68^Ga]Ga-(HE)_3_-Z_08698_-NOTA and [^68^Ga]Ga-Z_08698_-NODAGA, ^d^ [^68^Ga]Ga-Z_08698_-NOTA and [^68^Ga]Ga-(HE)_3_-Z_08698_-NODAGA, ^e^ [^68^Ga]Ga-Z_08698_-NOTA and [^68^Ga]Ga-Z_08698_-NODAGA, ^f^ [^68^Ga]Ga-(HE)_3_-Z_08698_-NODAGA and [^68^Ga]Ga-Z_08698_-NODAGA.

## Supplementary Materials

Supplementary materials can be found at https://www.mdpi.com/1422-0067/20/5/1080/s1.

## Abbreviations

CT	Computed Tomography
EDTA	Ethylenediaminetetraacetic Acid
EGFR	Epidermal Growth Factor Receptor
ESI-MS	Electrospray Ionization Mass Spectrometry
HER2	Human Epidermal Growth Factor Receptor 2
HER3	Human Epidermal Growth Factor Receptor 3
IPTG	Isopropyl β-D-1-thiogalactopyranoside
ITLC	Instant Thin Layer Chromatography
MES	2-(N-morpholino)-ethanesulfonic Acid
MIP	Maximum Intensity Projection
NODAGA	1-(1,3-carboxypropyl)-4,7-carboxymethyl-1,4,7-triazacyclononane
NOTA	1,4,7-triazacyclononane-N,N′,N′′-triacetic acid
PBS	Phosphate-Buffered Saline
PET	Positron Emission Tomography
RGB	Red, Green and Blue color scale
RP-HPLC	Reverse-Phase High Performance Liquid Chromatography
SPECT	Single Photon Emission Computed Tomography
SPR	Surface Plasmon Resonance
VTM	Variable Temperature Measurement
