# Supplementary material for: Molecular Design of HER3-Targeting Affibody Molecules: Influence of Chelator and Presence of HEHEHE-Tag on Biodistribution of 68Ga-Labeled Tracers
Source: Int J Mol Sci. 2019 Mar 2;20(5):1080. doi: 10.3390/ijms20051080 (PMC6429182; doi:10.3390/ijms20051080)
Supplement: Supplementary file 1 [file ijms-20-01080-s001.pdf]

International Journal of Molecular Sciences

MDPI

Supplementary Data

Molecular design of HER3-targeting affibody molecules: Influence of chelator and presence of HEHEHE-tag on biodistribution of ^{68}Ga-labeled tracers

Charles Dahlsson Leitao[1†], Sara S. Rinne[2†], Bogdan Mitran[2], Anzhelika Vorobyeva[3], Ken Andersson[1], Vladimir Tolmachev[3], Stefan Ståhl[1], John Löfblom[1], Anna Orlova[2,4 *]

[1]Department of Protein Science, School of Engineering Sciences in Chemistry, Biotechnology and Health, KTH Royal Institute of Technology, Stockholm, Sweden

[2]Department of Medicinal Chemistry, Uppsala University, Uppsala, Sweden

[3]Department of Immunology, Genetics and Pathology, Uppsala University, Uppsala, Sweden

[4]Science for Life Laboratory, Uppsala University, Sweden

[†]Equal Contribution

* Email: anna.orlova@ilk.uu.se, Telephone: +4618-471 5303, cell telephone: +46739922846;
Address: Dag Hammarskjöldsv 14C, 3tr, 751 83 Uppsala, Sweden

Supplementary Data

Results

HER3-binding affibody molecules Z_{08698} and $(HE)_3$-Z_{08698} were recombinantly produced in *E. coli* and coupled to maleimide derivatives of NOTA and NODAGA and subjected to reverse-phase high performance liquid chromatography (RP-HPLC) as a final step for remnant chelator removal and separation from unconjugated protein.

The purity, determined with RP-HPLC, exceeded 95% for all conjugates (Figure S1). Molecular mass was determined with ESI-MS (Table S1), observing no discrepancy between experimental and theoretical masses (Figure S2). The mass determination revealed non-processed N-terminal methionine for $(HE)_3$-Z_{08698}-NOTA and $(HE)_3$-Z_{08698}-NODAGA, owing to the presence of the (HE)3-tag at the N-terminus. Z_{08698}-NOTA and Z_{08698}-NODAGA exhibited additional peaks, which is likely the result of chelated metal contaminants. Thermal denaturation curves are shown in Figure S3 and the associated melting temperatures are presented in Table S1. Kinetic data from SPR analysis and associated K_D values are shown in Figure S4 and Table S1.

Table S1 Experimental molecular masses, affinities and melting temperatures of the conjugates. The theoretical molecular mass is in parenthesis. * Data published earlier [13, 24]

	Mw (Da)	K_D (pM, mean ± SD)	Tm (°C)
$(HE)_3$-Z_{08698}-NOTA*	8149.7 (8149.1)	55 ± 7.1	65.2
Z_{08698}-NOTA*	7219.6 (7219.1)	40 ± 1.5	63.8
$(HE)_3$-Z_{HER3}-NODAGA	8221.2 (8221.1)	38 ± 10	65.0
Z_{HER3}-NODAGA*	7291.5 (7291.2)	11 ± 0.6	64.3

Complete refolding was observed for each conjugate following thermal denaturation by comparison of spectra obtained at 20°C before and after denaturation (Figure S3). Kinetic data acquired from SPR analysis are presented in Table S1 as the average from duplicate injections. Representative sensorgrams with fitted curves for each conjugate are shown in Figure S4.

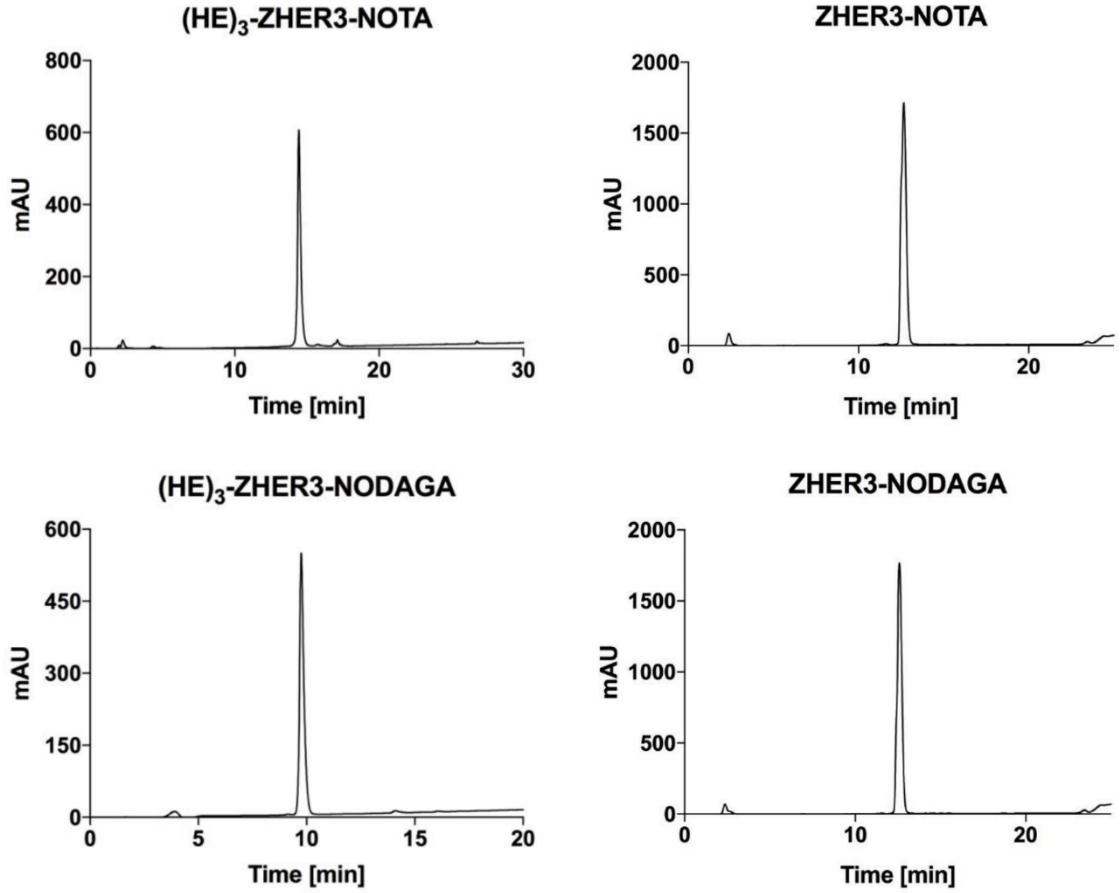

Figure S1 Evaluation of purity. Absorbance measurements at 220 nm from RP-HPLC was used to evaluate the purity of the four conjugates. Purity of $(HE)_3$-Z_{08698}-NOTA, Z_{08698}-NOTA and Z_{08698}-NODAGA was previously described [13,24].

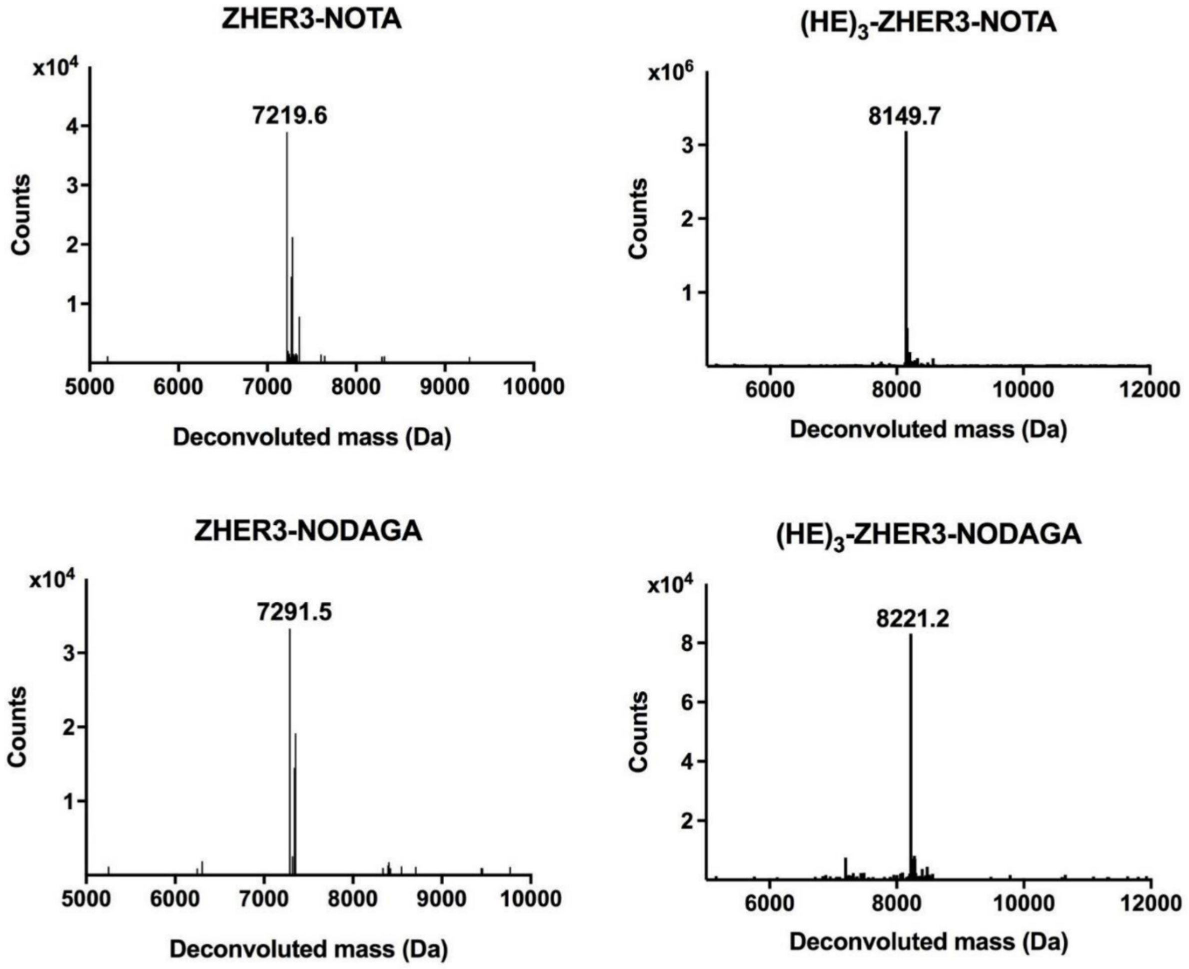

Figure S2: Determination of molecular mass. Observed peaks from ESI-MS were in agreement with the theoretical masses shown in Table S1. Molecular masses of $(HE)_3$-Z_{08698}-NOTA, Z_{08698}-NOTA and Z_{08698}-NODAGA were previously reported [13,24].

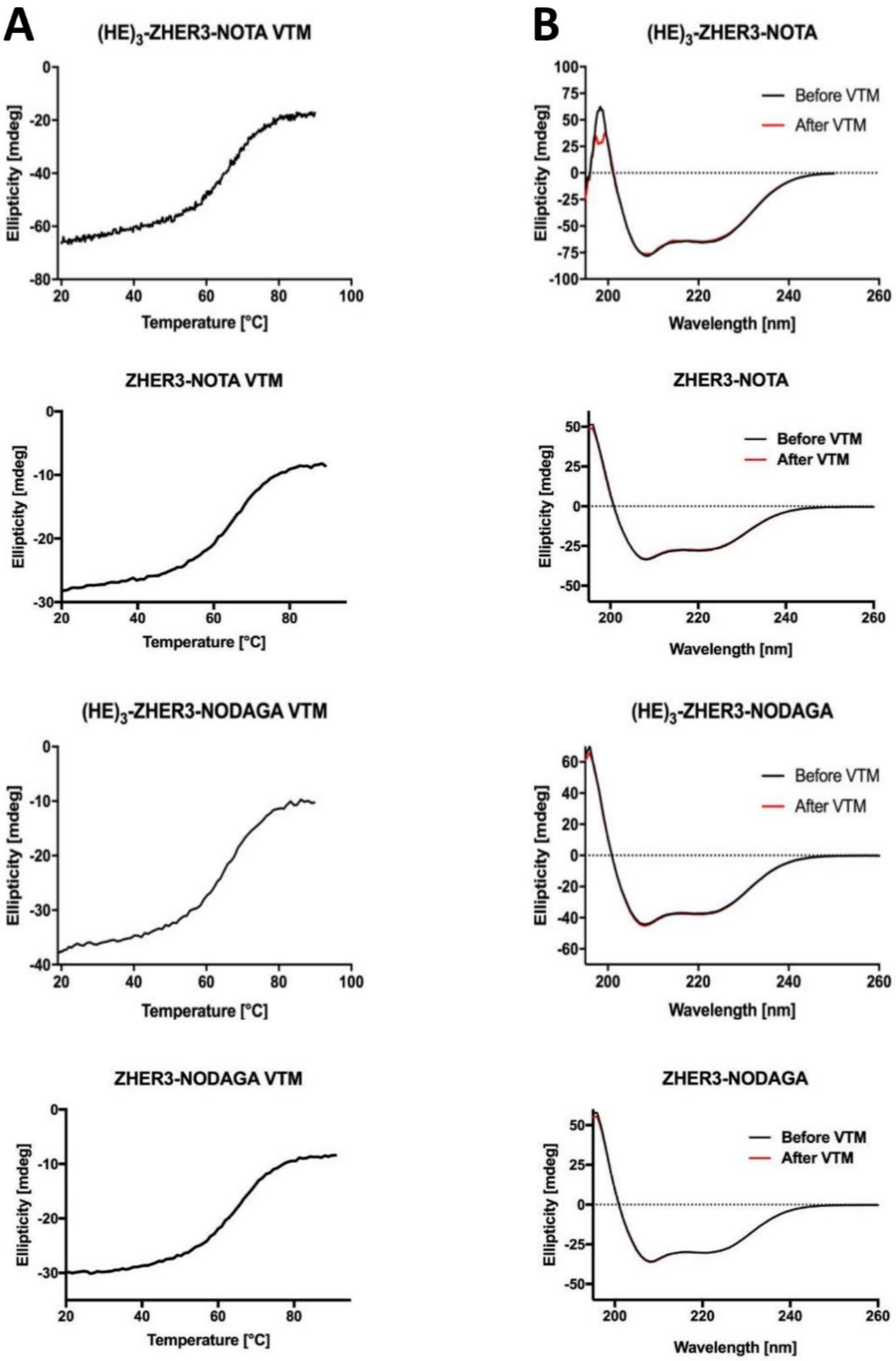

Figure S3 Analysis of thermal stability and refolding capacity. A) Thermal stability was evaluated, using variable temperature measurement (VTM), by observing the change in ellipticity at 221 nm while incrementally heating the sample from 20°C to 90°C. B) Superimposed circular dichroism spectra, measured at 20°C and in the range 195-260 nm, before and after thermal denaturation, demonstrating complete refolding capacity. Melting temperatures (T_m) were determined by fitting the curves using a Boltzmann Sigmoidal model. The determined T_m values for each conjugate are presented in Table S1. Thermal stability and refolding of $(HE)_3$-Z_{08698}-NOTA, Z_{08698}-NOTA and Z_{08698}-NODAGA were previously described [13,24].

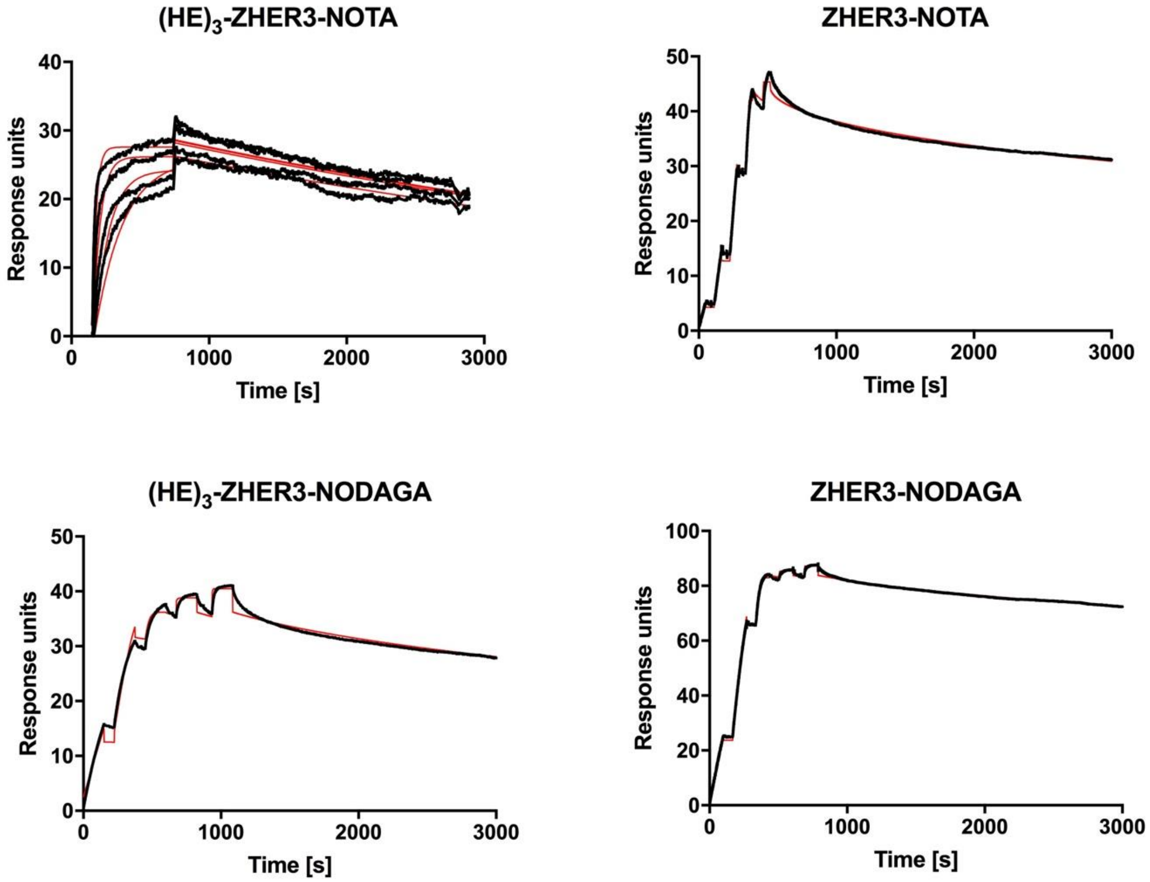

Figure S4: Representative experimental sensorgrams (black) with fitted curves (red) from SPR analysis. Immobilized human HER3 was subjected to five concentrations (3.125, 6.25, 12.5, 25 and 50 nM) of NODAGA-conjugated affibody in a single cycle. For NOTA-conjugated affibody, four concentrations (1.875, 3.75, 7.5 and 15 nM) were injected in a multi-cycle setup. Monovalent affinities, based on a Langmuir 1:1 model, are presented in Table S1. SPR analysis of (HE)$_3$-Z$_{08698}$-NOTA, Z$_{08698}$-NOTA and Z$_{08698}$-NODAGA was previously reported [13,24].

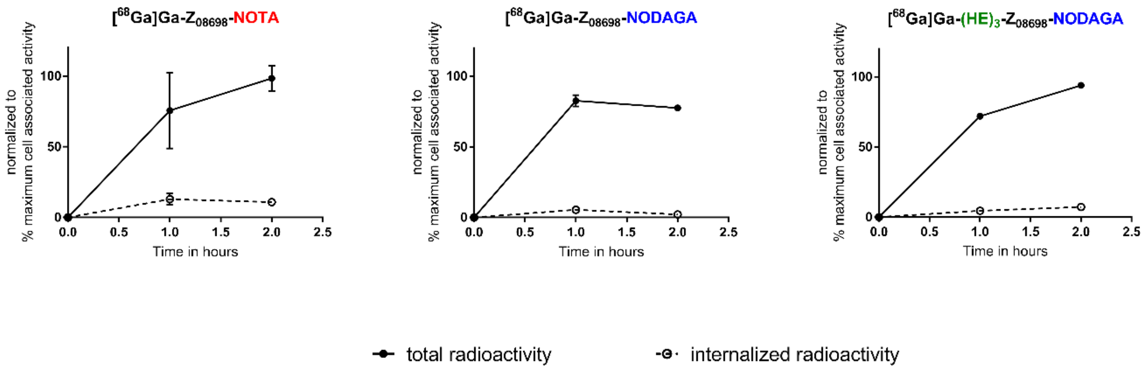

Figure S5: Cellular processing of [^{68}Ga]Ga-Z$_{08698}$-NOTA, [^{68}Ga]Ga-Z$_{08698}$-NODAGA, [^{68}Ga]Ga-(HE)$_3$-Z$_{08698}$-NODAGA on DU145 cells. Cells were continuously incubated with 0.1 nM of labeled construct at 37°C. Error bars may not be visible because they are smaller than the curve symbols.
